# Macronutrient quality and colorectal cancer outcomes: evidence from the PLCO screening trial

**DOI:** 10.3389/fnut.2025.1656275

**Published:** 2026-01-12

**Authors:** Dazhan Feng, Ling Xiang, Ke Wen, Yi Xiao, Haitao Gu, Linglong Peng, Yuxiang Luo, Yaxu Wang, Dengliang Liu, Zhihang Zhou, Qian Liu

**Affiliations:** 1 Department of Gastrointestinal Surgery, The Second Affiliated Hospital of Chongqing Medical University, Chongqing, China; 2 Department of Clinical Nutrition, The Second Affiliated Hospital of Chongqing Medical University, Chongqing, China; 3 Department of Radiation Oncology, Senior Department of Oncology, The Fifth Medical Center of PLA General Hospital, Beijing, China; 4 Erasmus University Medical Center, Rotterdam, Netherlands; 5 Department of Gastrointestinal Surgery, Chongqing Jiulongpo People’s Hospital, Chongqing, China; 6 Department of General Surgery, Xipeng Town Health Center of Jiulongpo District, Chongqing, China; 7 Department of Gastroenterology, The Second Affiliated Hospital of Chongqing Medical University, Chongqing, China; 8 Department of Dermatology and Cosmetic Medicine Center, The Second Affiliated Hospital of Chongqing Medical University, Chongqing, China

**Keywords:** macronutrient quality index, cancer prevention, epidemiology, colorectal cancer, cohort study

## Abstract

**Background:**

No previous study has assessed the relationship between macronutrient quality and colorectal cancer (CRC) incidence and mortality. Thus, to further explore the associations between macronutrient quality and CRC risk, we conducted a large prospective cohort study involving 101,709 people in the United States from the Prostate, Lung, Colorectal, and Ovarian (PLCO) Cancer Screening Trial.

**Methods:**

Our study population was derived from 154,887 adults aged 55 to 74 years who were recruited from 10 screening centers in the United States. The macronutrient quality index (MQI) was calculated based on dietary history questionnaire (DHQ). Cox regression analysis was utilized to calculate the hazard ratios (HRs) and 95% confidence intervals (CIs) of the associations between MQI and CRC incidence and mortality. We used subgroup analyses to identify potential effect modifiers. Sensitivity analysis was performed to ensure the study findings were robust.

**Results:**

During the study period, 1,100 colorectal cancer (CRC) diagnoses and 314 CRC-related deaths were recorded. Higher adherence to the MQI was significantly associated with reduced CRC risk, demonstrating a 22% lower incidence (HR Q4 vs. Q1: 0.78; 95% CI: 0.65–0.93; *p* = 0.006 for trend) and 38% lower mortality (HR Q4 vs. Q1: 0.62; 95% CI: 0.44–0.86; *p* = 0.001 for trend) in the highest vs. lowest quartiles. These associations were robust across sensitivity analyses. Subsite-specific analyses revealed pronounced protective effects for distal colon cancer incidence (36% reduction; HR: 0.64; 95% CI: 0.43–0.96; *p* = 0.010 for trend) and mortality (56% reduction; HR: 0.44; 95% CI: 0.19–1.01; *p* = 0.037 for trend), with significant mortality reductions also observed for proximal colon cancer (34%; HR: 0.66; 95% CI: 0.44–1.00; *p* = 0.031 for trend).

**Conclusion:**

Our findings suggest focusing on higher quality of macronutrient consumption may be an effective approach to reduce the risk of CRC in the US population.

## Introduction

Colorectal cancer (CRC) continues to impose a substantial public health burden in the United States, with projections forecasting over 152,000 new diagnoses and 52,000 CRC-related deaths by 2025 (1). This malignancy ranks second in incidence among women and third among men, underscoring its prevalence across both genders (2). The profound health and economic impacts of CRC reinforce the urgent need for innovative, evidence-based prevention strategies to mitigate its incidence and mortality rates.

Emerging evidence underscores the central role of dietary patterns as modifiable lifestyle factors in CRC etiology, with over 40% of cases and deaths attributable to suboptimal nutritional choices (3). The quality and source of macronutrients— proteins, carbohydrates, and fats—constitute critical dimensions of diet quality, exhibiting complex associations with CRC risk. Animal-derived low-quality proteins, containing saturated fats, cholesterol, and heterocyclic amines, may promote intestinal carcinogenesis by stimulating aberrant cell proliferation (4), whereas plant-based high-quality proteins, rich in fiber, antioxidants, and phytochemicals, may exert protective effects through anti-inflammatory and antioxidant mechanisms (5). Concurrently, carbohydrate and fat quality also play pivotal roles: high-glycemic-index carbohydrates may indirectly promote tumor growth via insulin fluctuations (6), while specific fatty acid profiles, such as *ω*-3 polyunsaturated fatty acids, are linked to reduced CRC risk (7). However, conventional research prioritizing single nutrients or total intake has overlooked macronutrient synergies and quality disparities, leading to conflicting conclusions (8).

To address these limitations, the macronutrient quality index (MQI) was developed as a multidimensional framework integrating nutrient ratios, fatty acid profiles, and food source quality to assess dietary patterns comprehensively (8, 9). While existing indices (e.g., food-group-based scoring systems) offer partial insights, they inadequately characterize macronutrient quality, particularly lacking CRC-specific outcome-oriented evaluation tools (10).

Leveraging data from the Prostate, Lung, Colorectal, and Ovarian (PLCO) Cancer Screening Trial, this study pioneers the application of MQI to investigate associations between global macronutrient quality and CRC incidence and mortality. Building on conventional risk assessments, we further dissected CRC outcomes by anatomical subsite—distinguishing proximal colon, distal colon, and rectal cancers—to evaluate whether macronutrient quality differentially influences site-specific carcinogenesis. By synthesizing energy source distributions, fatty acid quality, and food matrix effects, MQI aims to elucidate diet-cancer relationships beyond univariate nutrient analyses, providing evidence-based foundations for precision dietary intervention strategies tailored to CRC subsite heterogeneity.

## Methods

### Study design

The current analysis is a secondary analysis of the data from the PLCO Cancer Screening Trial, a large-scale randomized clinical trial initiated and funded by the National Cancer Institute (NCI) from 1993 to 2001. It is important to note that the PLCO trial itself possesses the characteristics of a prospective study. On this basis, the data utilized in this analysis are all systematically collected from participants during the course of this prospective research (11). This initiative recruited approximately 150,000 men and women aged 55–74 years across 10 screening centers nationwide. Participants were randomly assigned to either standard medical care (control arm) or enhanced cancer screening protocols (intervention arm) (12). The trial protocol underwent rigorous ethical review and received approval from institutional review boards at both NCI headquarters and participating study sites. All participants provided voluntary, informed consent prior to enrollment. Comprehensive methodological details of the PLCO trial design, including statistical power calculations and participant recruitment strategies, have been previously published (13, 14).

### Data collection and covariates assessment

In the PLCO trial, baseline demographic and lifestyle information was collected via self-reported surveys (Baseline Questionnaire, BQ). Our analysis focused on key variables including age, sex, race, marriage, smoking status, daily cigarette consumption, history of alcohol consumption, baseline body mass index (BMI), history of aspirin usage, history of diabetes, hypertension, colorectal diverticulitis/diverticulosis, colorectal polyps, and colorectal comorbidities such as Gardner’s syndrome, ulcerative colitis, Crohn’s disease, or familial polyposis, along with family history of CRC. BMI was calculated using the standard formula: weight (kg)/height^2^(m^2^). Dietary assessment occurred 3 years post-enrollment using the validated 137-item Dietary History Questionnaire (DHQ), a Food Frequency Questionnaire (FFQ) designed to evaluate portion sizes, consumption frequencies, and types of foods/supplements over the prior year. The DHQ’s accuracy was validated against the Eating at America’s Table Study, a 24-h dietary recall reference, demonstrating superior performance in quantifying absolute nutrient intake compared to established FFQs like the Block and Willett questionnaires (15).

### Population for analysis

Participants were further excluded based on the following criteria:

1. Participants who failed to complete the Baseline Questionnaire (BQ) were excluded (*n* = 4,918).
2. Responses to the Dietary History Questionnaire (DHQ) were considered invalid and led to participant exclusion under the following circumstances: failure to return the DHQ; lack of a completion date; completion after the participant’s death date; a high frequency of missing responses (≥8); or extremely high energy intake values (falling within the first or last percentile). A total of 38,462 participants were excluded based on these criteria.
3. Participants with a history of cancer prior to DHQ administration were excluded (*n* = 9,684).
4. Participants who withdrew from the PLCO cancer screening trial between enrollment and DHQ completion, due to outcome events, death, or loss to follow-up, were excluded (*n* = 114).

**Figure 1:**
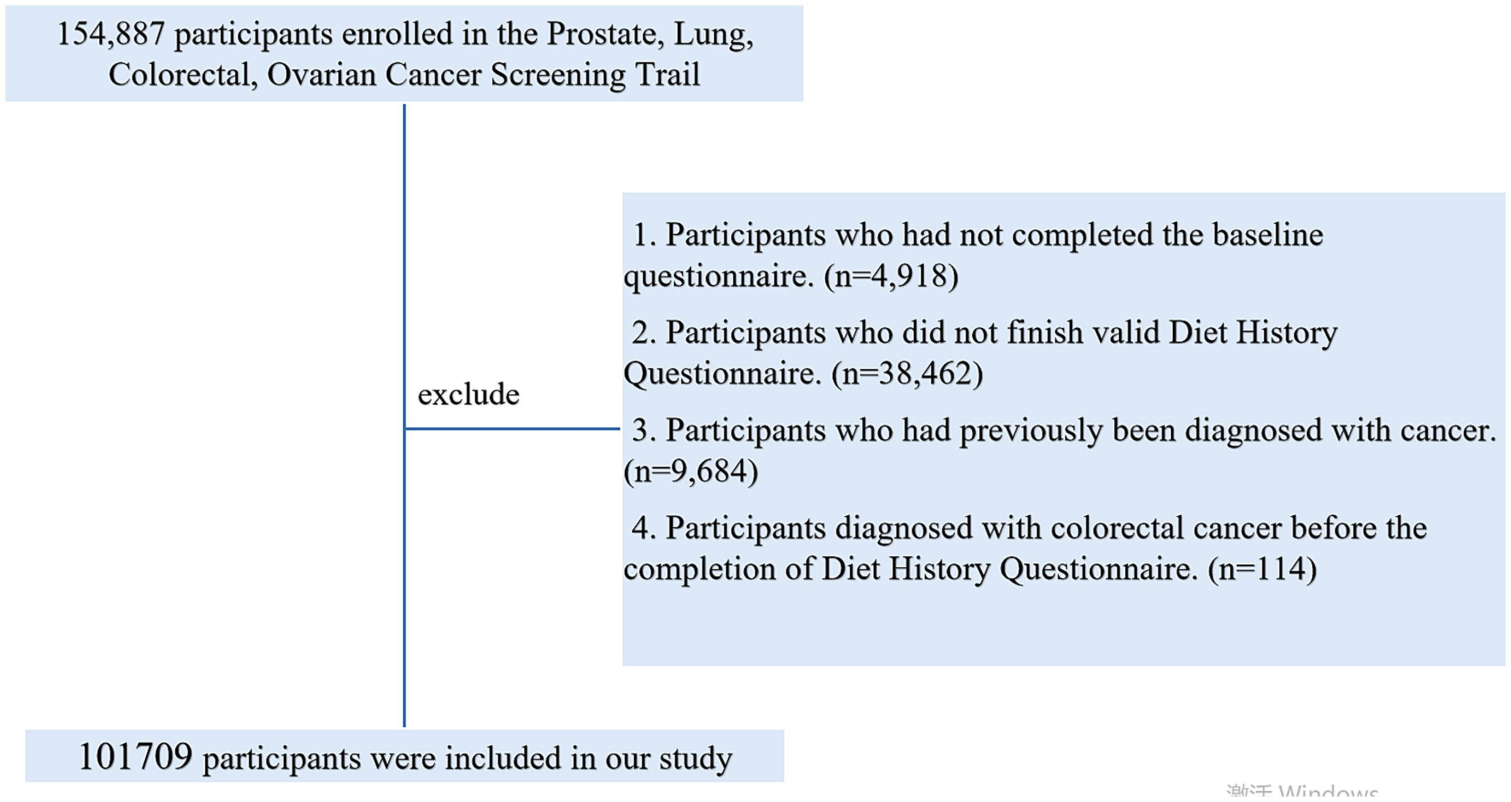
The flow chart of identifying eligible subjects. PLCO, Prostate, Lung, Colorectal, and Ovarian; BQ, Baseline questionnaire; DHQ, Diet history questionnaire.

Ultimately, as illustrated in Figure 1, a total of 101,709 participants met the inclusion criteria, comprising 52,250 females and 49,459 males.

### Calculation of MQI

The Macronutrient Quality Index (MQI) operates as a comprehensive dietary assessment tool, integrating three distinct sub-indices to evaluate macronutrient intake quality: the Carbohydrate Quality Index (CQI), Fat Quality Index (FQI), and Healthy Plate Protein Quality Index (HPPQI). Each sub-index contributes equally to the total MQI score, with higher scores indicating superior dietary quality. This methodology enables multidimensional evaluation of dietary patterns, capturing both nutrient composition and food source quality.

The CQI comprises four key components, each scored from 1 to 5 based on participant quintiles. First, glycemic index (GI) is reverse-scored to prioritize low-GI diets associated with reduced metabolic dysregulation risk. Second, total dietary fiber intake is directly scored, reflecting its role in promoting satiety and gut health. Third, the solid/liquid carbohydrate ratio differentiates whole food intake from processed beverages, with higher scores favoring solid carbohydrate sources. Fourth, the whole grain/total grain ratio emphasizes whole grain consumption over refined grains, aligning with evidence supporting whole grains for chronic disease prevention (16–20). The summed score of these four components yields a CQI ranging from 4 to 20.

The FQI serves as a fat quality indicator that assigns lower scores to diets high in inflammation-promoting fats while giving higher scores to those rich in physiologically beneficial unsaturated fats. This method ensures equitable consideration of all fatty acid types, providing a comprehensive evaluation of dietary lipid composition (17, 19). The FQI is computed using the following ratio:


$$\begin{array}{l} \mathrm{FQI}=(\text{Monounsaturated}+\text{Polyunsaturated})\\ /(\text{Saturated}+\text{Trans Fatty Acids})\;(\text{MUFA}+\text{PUFA})\\ /(\mathrm{SFA}+\mathrm{TFA}) \end{array}$$

The HPPQI assesses protein source quality via a ratio favoring seafood, poultry, legumes, and nuts—sources rich in high-quality protein, essential amino acids, and bioactive compounds—over red/processed meats and cheese (21, 22). This sub-index aligns with global dietary guidelines emphasizing plant-forward protein choices (23). The HPPQI is computed using the following ratio:


$$\begin{array}{l} \text{HPPQI}=(\text{seafood}+\text{poultry}+\text{pulses}+\text{nuts})\\ /(\mathrm{red}\;\text{and processed meats}+\text{cheese}) \end{array}$$

Finally, as each sub-index contributes equally to the MQI total score, participants are assigned quintile-based scores (1–5 points) for each sub-index. The MQI score is calculated by summing these sub-index scores, resulting in a total range of 3 to 15 (24). This framework allows for granular evaluation of dietary quality across macronutrient domains, providing deep insights into diet-health relationships. The methodology has been validated in large cohort studies, demonstrating its utility for assessing dietary patterns in relation to chronic disease outcomes (24, 25).

### Ascertainment of outcome events

In the PLCO trial, colorectal cancer (CRC) case ascertainment primarily utilized annual participant follow-up surveys mailed to surviving cohort members, which collected self-reported data on new cancer diagnoses. These self-reported CRC diagnoses underwent standardized medical verification through blinded adjudication of medical records by study physicians. Participant survival status was monitored via these annual surveys, with multiple follow-up attempts conducted for non-responders to ensure comprehensive data collection. Additional mortality surveillance was implemented through routine data linkages with the National Death Index (NDI), supplemented by examination of death certificates using ICD-9 coding to confirm causes of mortality. For anatomical classification of CRC cases, diagnoses were categorized according to International Classification of Diseases for Oncology, Second Edition (ICD-O2) codes, distinguishing between proximal colon (C180-C185), distal colon (C186-C187), and rectal (C199-C209) malignancies. Cases with ambiguous or overlapping subsite designations (C188, C189, C212, C218) were systematically excluded from subsite-specific analyses to maintain analytical clarity.

### Statistical analysis

To address missing covariate data in this analysis, a systematic imputation approach was applied based on missingness proportion and variable type. For categorical variables with <5% missingness (e.g., marital status, race, smoking status, daily cigarette consumption, aspirin use history, hypertension history, diabetes history, colorectal diverticulitis/diverticulosis history, colorectal polyps history, colorectal comorbidities history, family cancer history, family CRC history), missing values were filled using the mode. Continuous variables with <5% missing data (including baseline BMI, BMI change, and pack-years of cigarette smoking) were filled using the median (26). Detailed imputation specifications for each variable and corresponding missingness proportions are provided in Supplementary Table 1.

**Figure 2:**
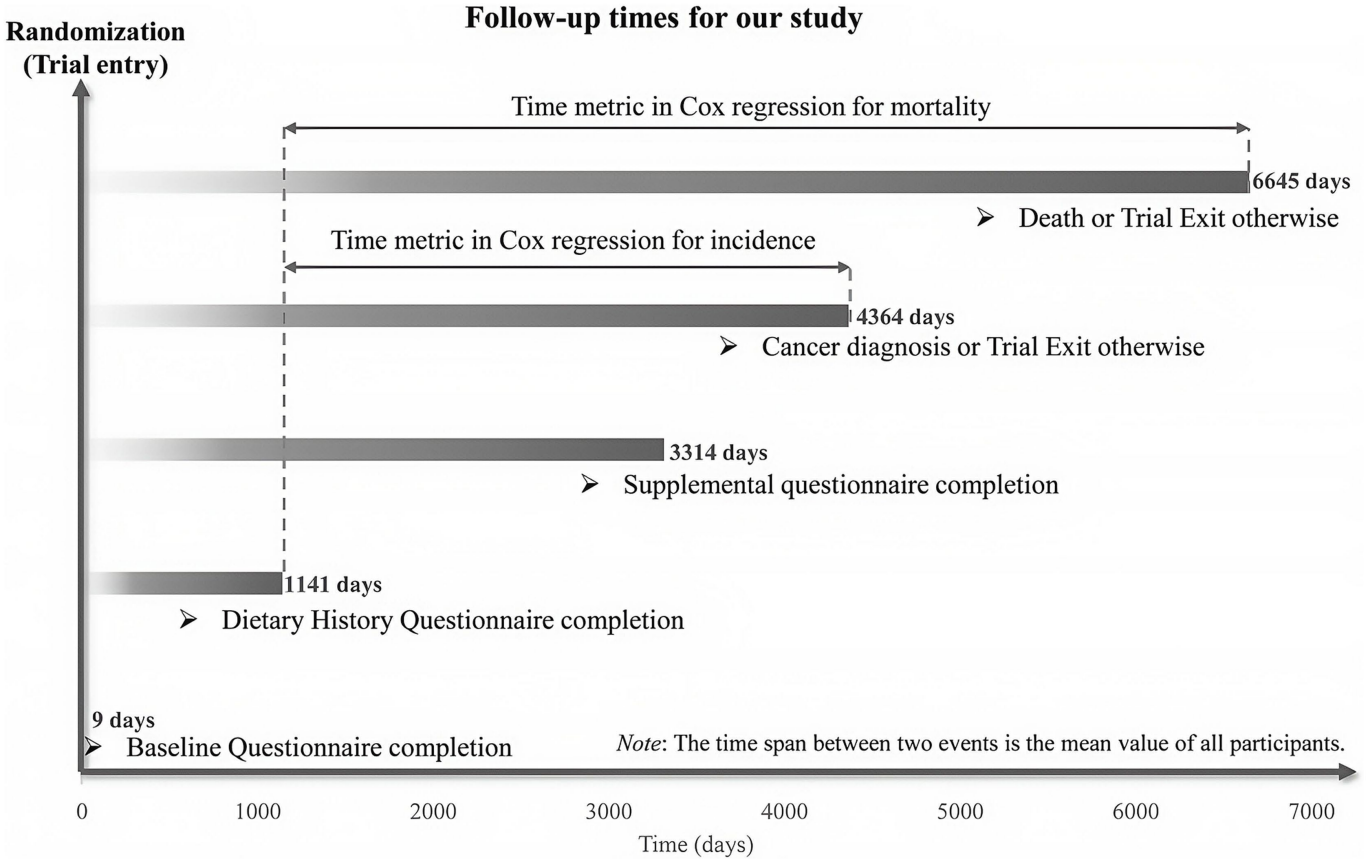
The timeline and follow-up scheme of our study.

This study measured time-to-CRC-event as days from DHQ completion to CRC diagnosis/related death. Follow-up for primary outcomes commenced at DHQ completion and terminated at first occurrence of CRC diagnosis, death, loss to follow-up, or December 31, 2009 (cancer incidence follow-up conclusion). Secondary mortality outcomes were tracked until 2018 per PLCO website specifications (Figure 2).^1^ Associations between MQI and outcomes were analyzed using Cox proportional hazards models, with follow-up duration as time metric, to estimate HRs and 95% CIs. MQI was quartile-stratified (first quartile = referent) and continuous variables derived from quartile medians tested linear trends (significance: *p*-values) (27). Cox models incorporated two adjustment sets: Model 1 adjusted for demographic factors (sex, age, race and marital status), and Model 2 further adjusted for lifestyle/clinical factors (BMI, smoking status, daily cigarette use, history of alcohol consumption, history of hypertension, history of diabetes, history of colorectal comorbidities, polyps and diverticulitis/diverticulosis, aspirin use and family history of CRC) and trial group. Nonlinear relationships between MQI and CRC incidence/mortality were evaluated using restricted cubic splines (RCS), referencing median MQI values. Nonlinearity was determined by testing second-term spline regression coefficients against zero (28). Parallel analyses were meticulously conducted for the anatomical subsites of CRC, specifically categorizing them into proximal colon cancer, distal colon cancer, and rectal cancer. However, it is important to note that due to the extremely small number of patients whose tumor anatomical locations could not be precisely determined, these cases were excluded from the aforementioned categorical statistical analyses to ensure the accuracy and reliability of the results.

To evaluate potential modification effects of key factors on the associations between MQI and both CRC incidence and mortality, we conducted predefined subgroup analyses across multiple categorical strata. Subgroup stratification was implemented based on the following: demographic characteristics: age (>65 vs. ≤65 years), sex (male vs. female), race (White vs. non-White), marital status (married vs. unmarried); health conditions: diabetes status (yes vs. no), hypertension status (yes vs. no), baseline BMI (≤30 vs. >30 kg/m^2^); family and medical history: family history of CRC (absent vs. present), history of colorectal diverticulitis/diverticulosis (yes vs. no), history of colorectal comorbidities (yes vs. no), history of colorectal polyps (yes vs. no); lifestyle factors: smoking status (never vs. current/former), history of alcohol consumption (yes vs. no), aspirin use (yes vs. no), daily cigarette consumption (0 vs. 1–20 vs. >20 cigarettes). Interaction *p*-values were calculated to detect any spurious subgroup effects by comparing models with and without interaction terms. This approach allowed us to assess the heterogeneity of effects across different subgroups for both CRC incidence and mortality outcomes while maintaining statistical rigor.

To enhance the robustness of the findings, we conducted some sensitivity analyses (27, 29, 30):

1. To address potential reverse causality, we conducted sensitivity analyses by excluding cases occurring within the first 2 years of follow-up.
2. Individuals with extreme energy intake (energy intake >4,000 kcal/day or <500 kcal/day) were excluded.
3. Individuals with extreme BMI values (the lowest 1% and the highest 1%) were excluded.
4. To improve the statistical power of the study, pack-years of smoking were adjusted instead of daily cigarette consumption (ranging from 0 to 20).
5. Individuals with diabetes, colorectal diverticulitis/diverticulosis or colorectal co-morbidity (Ulcerative colitis, Crohn’s disease, Gardner’s syndrome, or familial polyposis) were excluded.

All statistical analyses were carried out using R software version 4.3.1, with two-tailed *p* < 0.05 as the level of statistical significance.

## Results

### Participant baseline features

In this study, the MQI data were calculated based on the collected baseline dietary data. Participants were categorized into four quartiles based on their MQI values: Quartile 1 (3–7), Quartile 2 (8–9), Quartile 3 (10–11), and Quartile 4 (12–15). Table 1 shows that participants in Q4 (the highest quartile) were more likely to be female and non-White, but less likely to be married, users of aspirin, or be diagnosed with diabetes or hypertension. Moreover, participants in Q4 are less likely to have lower smoking intensity and have a lower and less variable BMI.

**Table 1:** Baseline characteristics of study population according to MQI.

Characteristics	Overall	Quartiles of overall MQI			
		Quartile 1	Quartile 2	Quartile 3	Quartile 4
Number of participants	101,709	36,197	23,765	21,080	20,667
Age	62.40 ± 5.28	61.94 ± 5.19	62.41 ± 5.27	62.68 ± 5.29	62.93 ± 5.37
Sex					
Male	49,459 (48.63%)	21,281 (58.79%)	11,383 (47.90%)	8,956 (42.49%)	7,839 (37.93%)
Female	52,250 (51.37%)	14,916 (41.21%)	12,382 (52.10%)	12,124 (57.51%)	12,828 (62.07%)
Race					
White	94,023 (92.44%)	34,462 (95.21%)	22,057 (92.81%)	19,070 (90.46%)	18,434 (89.20%)
Non-white	7,686 (7.56%)	1,735 (4.79%)	1,708 (7.19%)	2,010 (9.54%)	2,233 (10.80%)
Marriage					
Married	79,788 (78.45%)	28,642 (79.13%)	18,930 (79.65%)	16,521 (78.37%)	15,695 (75.94%)
Unmarried	21,921 (21.55%)	7,555 (20.87%)	4,835 (20.35%)	4,559 (21.63%)	4,972 (24.06%)
Diabetes history					
No	94,907 (93.31%)	33,652 (92.97%)	22,163 (93.26%)	19,664 (93.28%)	19,428 (94.00%)
Yes	6,802 (6.69%)	2,545 (7.03%)	1,602 (6.74%)	1,416 (6.72%)	1,239 (6.00%)
Aspirin use history					
No	53,927 (53.02%)	19,152 (52.91%)	12,565 (52.87%)	11,145 (52.87%)	11,065 (53.54%)
Yes	47,782 (46.98%)	17,045 (47.09%)	11,200 (47.13%)	9,935 (47.13%)	9,602 (46.46%)
Family history of colorectal cancer					
No	88,910 (87.42%)	31,563 (87.20%)	20,825 (87.63%)	18,428 (87.42%)	18,094 (87.55%)
Yes	10,306 (10.13%)	3,597 (9.94%)	2,361 (9.93%)	2,187 (10.37%)	2,161 (10.46%)
Possibly	2,493 (2.45%)	1,037 (2.86%)	579 (2.44%)	465 (2.21%)	412 (1.99%)
Diverticulitis/Diverticulosis history					
No	94,886 (93.29%)	33,969 (93.84%)	22,194 (93.39%)	19,526 (92.63%)	19,197 (92.89%)
Yes	6,823 (6.71%)	2,228 (6.16%)	1,571 (6.61%)	1,554 (7.37%)	1,470 (7.11%)
Colorectal comorbidities history					
No	100,353 (98.67%)	35,712 (98.66%)	23,437 (98.62%)	20,792 (98.63%)	20,412 (98.77%)
Yes	1,356 (1.33%)	485 (1.34%)	328 (1.38%)	288 (1.37%)	255 (1.23%)
Colorectal polyp history					
No	94,944 (93.35%)	33,871 (93.57%)	22,196 (93.40%)	19,616 (93.06%)	19,261 (93.20%)
Yes	6,765 (6.65%)	2,326 (6.43%)	1,569 (6.60%)	1,464 (6.94%)	1,406 (6.80%)
Hypertension history					
No	68,678 (67.52%)	24,044 (66.43%)	15,848 (66.69%)	14,304 (67.86%)	14,482 (70.07%)
Yes	33,031 (32.48%)	12,153 (33.57%)	7,917 (33.31%)	6,776 (32.14%)	6,185 (29.93%)
Family history of cancer					
No	44,876 (44.12%)	16,183 (44.71%)	10,491 (44.14%)	9,204 (43.66%)	8,998 (43.54%)
Yes	56,833 (55.88%)	20,014 (55.29%)	13,274 (55.86%)	11,876 (56.34%)	11,669 (56.46%)
Arm					
Intervention	51,778 (50.91%)	18,225 (50.35%)	12,016 (50.56%)	10,664 (50.59%)	10,873 (52.61%)
Control	49,931 (49.09%)	17,972 (49.65%)	11,749 (49.44%)	10,416 (49.41%)	9,794 (47.39%)
Smoking status					
No	48,562 (47.75%)	15,515 (42.86%)	11,411 (48.02%)	10,575 (50.17%)	11,061 (53.52%)
Current/former	53,147 (52.25%)	20,682 (57.14%)	12,354 (51.98%)	10,505 (49.83%)	9,606 (46.48%)
Body mass index at baseline (kg/m2)	27.22 ± 4.79	27.93 ± 4.83	27.39 ± 4.80	26.90 ± 4.71	26.13 ± 4.54
Weight fluctuation^a^	2.88 ± 0.76	3.00 ± 0.75	2.91 ± 0.75	2.83 ± 0.75	2.70 ± 0.74
Smoking pack-years	17.65 ± 26.59	21.86 ± 29.73	17.67 ± 26.48	15.30 ± 24.25	12.66 ± 21.52
Daily cigarette consumption					
0	48,666 (47.85%)	15,564 (43.00%)	11,431 (48.10%)	10,593 (50.25%)	11,078 (53.60%)
1–20	33,203 (32.65%)	11,886 (32.84%)	7,713 (32.46%)	6,961 (33.02%)	6,643 (32.14%)
>20	19,840 (19.51%)	8,747 (24.16%)	4,621 (19.44%)	3,526 (16.73%)	2,946 (14.25%)
History of alcohol consumption					
No	27,741 (27.27%)	9,455 (26.12%)	6,296 (26.49%)	5,833 (27.67%)	6,157 (29.79%)
Yes	73,968 (72.73%)	26,742 (73.88%)	17,469 (73.51%)	15,247 (72.33%)	14,510 (70.21%)

Descriptive statistics are presented as (mean ± standard deviation) and number (percentage) for continuous and categorical.

a Weight fluctuation was defined as the participant’s baseline weight minus weight at age 20.

### Association between CRC incidence and MQI

**Figure 3:**
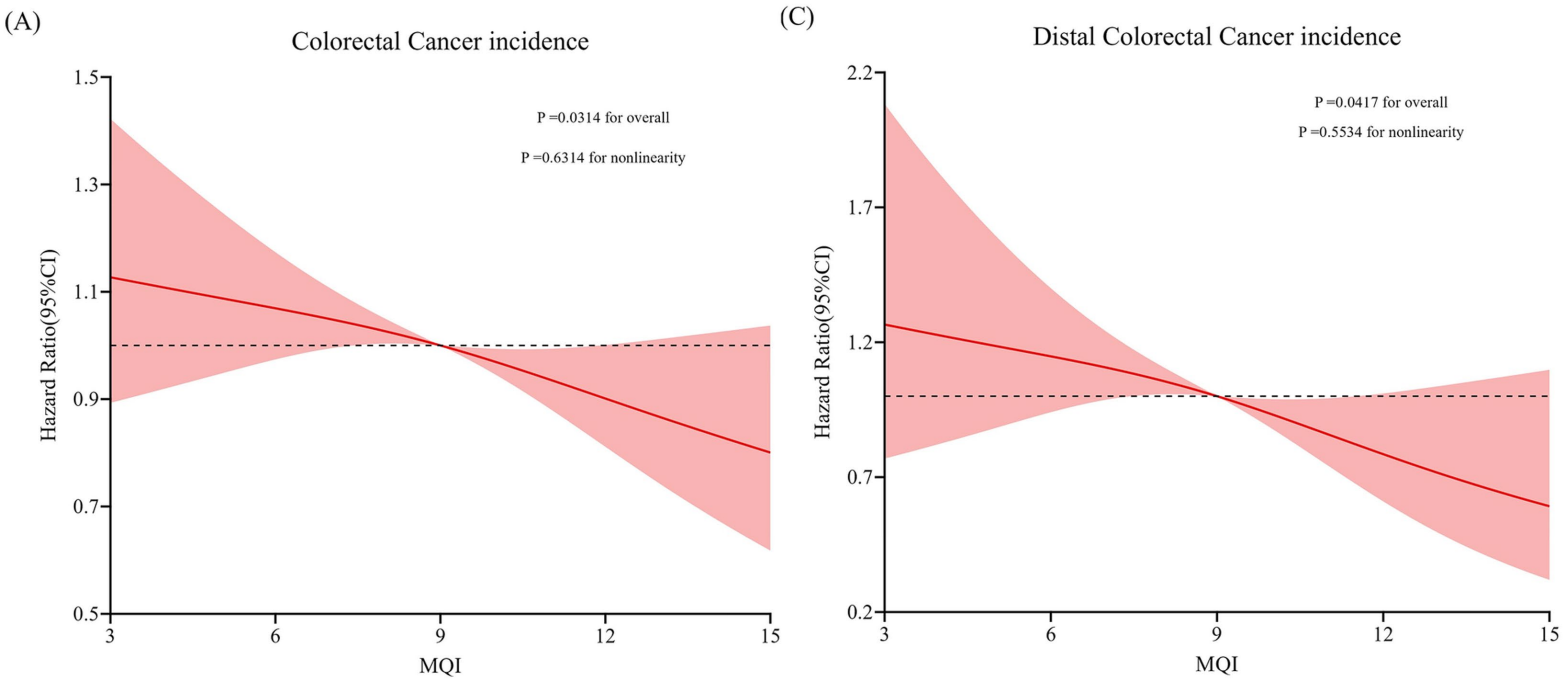
RCS model on the association of MQI with the CRC incidence **(A)** and distal CRC incidence **(C)**. Hazard ratio was adjusted for age (years), sex (male, female), race (White and non-White), marital status (married, unmarried), smoking status (never, currently/ever), number of cigarettes smoked (0, 1–20, > 20 cigarettes/day), history of alcohol consumption (yes, no), history of colorectal diverticulitis/ diverticulosis (yes, no), history of colorectal comorbidities (yes, no), history of colorectal polyps (yes, no), body mass index (kg/m2), trail arm (intervention, control), aspirin use (yes, no), history of diabetes (yes, no), history of hypertension (yes, no) and family history of CRC (yes, no).

During the median follow-up period of 8.82 years (896,103 person-years), we observed 1,100 incident CRC cases, comprising 648 proximal colon cancers, 226 distal colon cancers, 204 rectal cancers and 22 unknown anatomical location CRC cases. This yielded an overall incidence rate of 12.28 cases per 10,000 person-years. As illustrated in Table 2, multivariate Cox proportional hazards regression analysis, with comprehensive adjustment for potential confounders, demonstrated a significant inverse association between MQI and CRC incidence (HR for Q4 vs. Q1: 0.78; 95% CI: 0.65–0.93; *p* = 0.006 for trend). Subsite-specific analyses revealed distinct risk patterns: A robust protective effect of higher MQI was evident for distal colon cancer (HR Q4 vs. Q1: 0.64; 95% CI: 0.63–0.96; *p* = 0.010 for trend), while no significant associations were detected for proximal colon or rectal cancers (both *p* > 0.05 for trend). These findings were corroborated by restricted cubic spline (RCS) modeling, which identified linear relationships between increasing MQI and decreasing incidence rates for both overall CRC and distal colon cancer (Figure 3).

**Table 2:** Hazard ratios of the association between MQI and CRC incidence.

Quartiles of MQI	Cases	Person-years	Incidence rate per 10,000 person-years (95% confidence interval)	Hazard ratio (95% confidence interval) by MQI		
				Unadjusted	Model 1^a^	Model 2^b^
Colorectal cancer						
Quartile 1	420	315,467.1	13.31 (12.10,14.65)	1.000 (reference)	1.000 (reference)	1.000 (reference)
Quartile 2	268	209,823.5	12.77 (11.33,14.40)	0.96 (0.82,1.12)	0.95 (0.82,1.11)	0.97 (0.83,1.13)
Quartile 3	227	187,316.0	12.12 (10.64,13.80)	0.91 (0.78,1.07)	0.89 (0.76,1.05)	0.92 (0.78,1.09)
Quartile 4	185	183,502.4	10.08 (8.73,11.64)	0.76 (0.64,0.90)	0.73 (0.62,0.88)	0.78 (0.65,0.93)
p for trend				0.002	0.001	0.006
Proximal colon cancer						
Quartile 1	243	315,467.1	7.70 (6.79,8.73)	1.000 (reference)	1.000 (reference)	1.000 (reference)
Quartile 2	148	209,823.5	7.05 (6.01,8.28)	0.89 (0.72,1.09)	0.90 (0.73,1.11)	0.90 (0.73,1.11)
Quartile 3	138	187,316.0	7.37 (6.24,8.70)	0.91 (0.74,1.12)	0.93 (0.75,1.15)	0.93 (0.76,1.16)
Quartile 4	119	183,502.4	6.48 (5.42,7.76)	0.78 (0.63,0.98)	0.81 (0.65,1.02)	0.83 (0.66,1.03)
p for trend				0.161	0.041	0.120
Distal colon cancer						
Quartile 1	96	315,467.1	3.04 (2.49,3.72)	1.000 (reference)	1.000 (reference)	1.000 (reference)
Quartile 2	59	209,823.5	2.81 (2.18,3.63)	0.93 (0.67,1.28)	0.92 (0.66,1.27)	0.94 (0.67,1.30)
Quartile 3	37	187,316.0	1.98 (1.43,2.72)	0.65 (0.45,0.95)	0.63 (0.43,0.93)	0.66 (0.45,0.97)
Quartile 4	34	183,502.4	1.85 (1.33,2.59)	0.61 (0.41,0.90)	0.59 (0.39,0.87)	0.64 (0.43,0.96)
p for trend				0.004	0.002	0.010
Rectal cancer						
Quartile 1	76	315,467.1	2.41 (1.93,3.01)	1.000 (reference)	1.000 (reference)	1.000 (reference)
Quartile 2	55	209,823.5	2.62 (2.01,3.41)	1.09 (0.77,1.54)	1.15 (0.81,1.63)	1.18 (0.83,1.67)
Quartile 3	45	187,316.0	2.40 (1.80,3.21)	1.00 (0.69,1.44)	1.08 (0.74,1.57)	1.12 (0.77,1.63)
Quartile 4	28	183,502.4	1.53 (1.06,2.21)	0.63 (0.41,0.98)	0.70 (0.45,1.08)	0.75 (0.48,1.17)
p for trend				0.054	0.157	0.293

a Model 1 was adjusted with age (continuous), sex (male, female), race (White, non-White) and marital status (married, unmarried).

b Model 2 was adjusted for model 1 plus BMI at baseline (continuous), trail arm (intervention, control), smoking status (never, current or former), daily cigarette consumption (0, 1–20, >20), history of alcohol consumption (yes, no), aspirin use (no, yes), family history of CRC (no, yes, possibly), history of hypertension (no, yes), history of diabetes (no, yes), history of colorectal diverticulitis/diverticulosis (no, yes), history of colorectal comorbidities (no, yes) and history of colorectal polyp (no, yes).

Subgroup analysis revealed a significant interaction (*p* < 0.05) between colorectal polyps history in modulating the negative association between MQI and lung cancer incidence. This finding suggests that a higher MQI exerts a stronger protective effect in reducing the incidence of CRC among individuals without colorectal polyps history (HR Q4 vs. Q1: 0.78; 95% CI: 0.65, 0.94; *p* = 0.017 for trend) compared to those with colorectal polyps history (HR Q4 vs. Q1: 0.80; 95% CI: 0.44, 1.45; *p* = 0.138 for trend). In contrast, no significant differences in the negative association between MQI and CRC incidence were observed across subgroups defined by demographic characteristics (sex, race, marital status), health conditions (diabetes, hypertension, baseline BMI), family and medical history (family history of CRC, history of colorectal diverticulitis/diverticulosis and comorbidities), and lifestyle factors (aspirin use, smoking status, daily cigarette consumption and history of alcohol consumption; all interaction *p*-values > 0.05), as detailed in Supplementary Table 2. Furthermore, to ensure the reliability of the research findings regarding the relationship between the MQI and the incidence of CRC, we conducted sensitivity analyses. Given the potential interference of reverse causality, we first excluded CRC cases diagnosed within the first 2 years of follow-up. Subsequently, we further adjusted for potential confounders such as extreme values of energy intake and BMI. After this series of analyses, the results presented in Table 3 demonstrate that the association between MQI and CRC incidence remains robust.

**Table 3:** The sensitivity analyses between MQI and CRC incidence.

Categories	HR^e^ (Quartile 4 vs Quartile 1, 95% CI)	p for trend
Exclude extreme energy intake^a^	0.78 (0.64,0.94)	0.006
Exclude extreme BMI^b^	0.78 (0.65,0.93)	0.006
Excluding patients diagnosed within 2 years	0.75 (0.61,0.92)	0.005
Replace the indicator of cigarettes smoked^c^	0.78 (0.65,0.93)	0.007
Excluding patients with diabetes, diverticulitis/diverticulosis or colorectal co-morbidity^d^	0.81 (0.67,0.98)	0.024

a Extreme energy intake was defined as energy intake >4,000 kcal/day or <500 kcal/day.

b BMI defined as body mass index at baseline (kg/m^2^).

c Adjusting for pack-years of smoking (continuous) instead of daily cigarette consumption (0, 1–20, or >20).

d Colorectal co-morbidity included Ulcerative colitis, Crohn’s disease, Gardner’s syndrome, or familial polyposis.

e HR was adjusted for age (years), sex (male, female), race (White and non-White), marital status (married, unmarried), smoking status (never, currently/ever), number of cigarettes smoked (0, 1–20, > 20 cigarettes/day), history of alcohol consumption (yes, no), history of colorectal diverticulitis/ diverticulosis (yes, no), history of colorectal comorbidities (yes, no), history of colorectal polyps (yes, no), body mass index (kg/m^2^), trail arm (intervention, control), aspirin use (yes, no), history of diabetes (yes, no), history of hypertension (yes, no) and family history of CRC (no, yes).

### Association between CRC mortality and MQI

**Figure 4:**
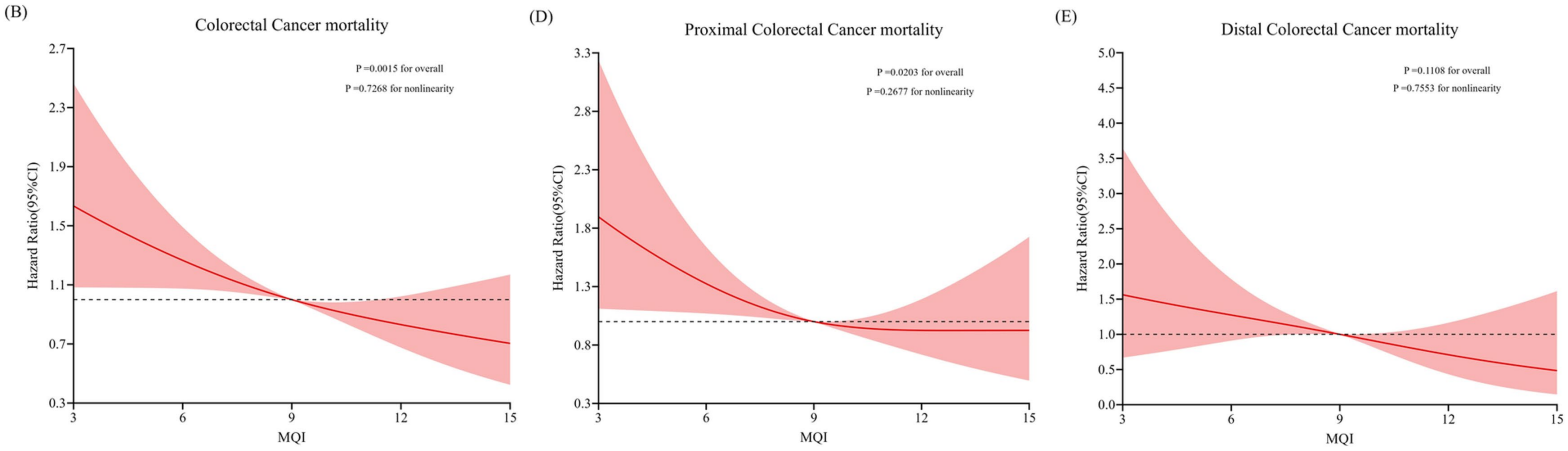
RCS model on the association of MQI with the CRC mortality **(B)**, proximal CRC mortality **(D)** and distal CRC mortality **(E)**. Hazard ratio was adjusted for age (years), sex (male, female), race (White and non-White), marital status (married, unmarried), smoking status (never, currently/ever), number of cigarettes smoked (0, 1–20, > 20 cigarettes/day), history of alcohol consumption (yes, no), history of colorectal diverticulitis/diverticulosis (yes, no), history of colorectal comorbidities (yes, no), history of colorectal polyps (yes, no), body mass index (kg/m^2^), trail arm (intervention, control), aspirin use (yes, no), history of diabetes (yes, no), history of hypertension (yes, no) and family history of CRC (yes, no).

During the median follow-up period of 15.07 years (1,532,681 person-years), we observed 314 deaths attributed to CRC, comprising 184 proximal colon cancer cases, 71 distal colon cancer cases, 54 rectal cancer cases and 5 unknown anatomical location cases. This yielded an overall mortality rate of 2.05 cases per 10,000 person-years. As illustrated in Table 4, multivariate Cox proportional hazards regression analysis, with comprehensive adjustment for potential confounders, demonstrated a significant inverse association between MQI and CRC mortality (HR for Q4 vs. Q1: 0.62; 95% CI: 0.44–0.86; *p* = 0.001 for trend). Subsite-specific analyses revealed distinct risk patterns: Robust protective effects of higher MQI were evident for proximal colon cancer (HR Q4 vs. Q1: 0.66; 95% CI: 0.44–1.00; *p* = 0.031 for trend) and distal colon cancer (HR Q4 vs. Q1: 0.44; 95% CI: 0.19–1.01; *p* = 0.037 for trend), while no significant association was detected for rectal cancers (*p* > 0.05 for trend). The RCS model revealed linear relationships between MQI and the mortality of overall CRC and proximal colon cancer (Figure 4).

**Table 4:** Hazard ratios of the association between MQI and CRC mortality.

Quartiles of MQI	Cases	Person-years	Incidence rate per 10,000 person-years (95% confidence interval)	Hazard ratio (95% confidence interval) by MQI		
				Unadjusted	Model 1^a^	Model 2^b^
Colorectal cancer						
Quartile 1	138	531,736.2	2.60 (2.20,3.07)	1.000 (reference)	1.000 (reference)	1.000 (reference)
Quartile 2	73	358,224.9	2.04 (1.62,2.56)	0.79 (0.60,1.05)	0.78 (0.59,1.04)	0.80 (0.60,1.06)
Quartile 3	54	323,083.8	1.67 (1.28,2.18)	0.65 (0.48,0.89)	0.63 (0.46,0.86)	0.66 (0.48,0.91)
Quartile 4	49	319,636.1	1.53 (1.16,2.03)	0.60 (0.43,0.83)	0.56 (0.40,0.78)	0.62 (0.44,0.86)
p for trend				0.001	<0.001	0.001
Proximal colon cancer						
Quartile 1	81	531,736.2	1.52 (1.23,1.89)	1.000 (reference)	1.000 (reference)	1.000 (reference)
Quartile 2	38	358,224.9	1.06 (0.77,1.46)	0.70 (0.48,1.03)	0.68 (0.46,1.00)	0.69 (0.47,1.02)
Quartile 3	32	323,083.8	0.99 (0.70,1.40)	0.66 (0.44,0.99)	0.62 (0.41,0.93)	0.64 (0.42,0.97)
Quartile 4	33	319,636.1	1.03 (0.74,1.45)	0.69 (0.46,1.03)	0.62 (0.41,0.94)	0.66 (0.44,1.00)
p for trend				0.043	0.015	0.031
Distal colon cancer						
Quartile 1	33	531,736.2	0.62 (0.44,0.87)	1.000 (reference)	1.000 (reference)	1.000 (reference)
Quartile 2	19	358,224.9	0.53 (0.34,0.83)	0.86 (0.49,1.51)	0.89 (0.50,1.56)	0.92 (0.52,1.63)
Quartile 3	12	323,083.8	0.37 (0.21,0.65)	0.61 (0.31,1.17)	0.62 (0.32,1.21)	0.69 (0.35,1.35)
Quartile 4	7	319,636.1	0.22 (0.11,0.45)	0.36 (0.16,0.81)	0.37 (0.16,0.84)	0.44 (0.19,1.01)
p for trend				0.007	0.010	0.037
Rectal cancer						
Quartile 1	21	531,736.2	0.39 (0.26,0.60)	1.000 (reference)	1.000 (reference)	1.000 (reference)
Quartile 2	15	358,224.9	0.42 (0.25,0.69)	1.06 (0.55,2.06)	1.07 (0.55,2.08)	1.10 (0.56,2.14)
Quartile 3	10	323,083.8	0.31 (0.17,0.57)	0.78 (0.37,1.66)	0.77 (0.36,1.65)	0.82 (0.38,1.76)
Quartile 4	8	319,636.1	0.25 (0.13,0.49)	0.63 (0.28,1.43)	0.61 (0.27,1.40)	0.69 (0.30,1.58)
p for trend				0.222	0.196	0.318

a Model 1 was adjusted with age (continuous), sex (male, female), race (White, non-White) and marriage (married, unmarried).

b Model 2 was adjusted for model 1 plus BMI at baseline (continuous), trail arm (intervention, control), smoking status (never, current or former), daily cigarette consumption (0, 1–20, >20), history of alcohol consumption (yes, no), aspirin use (no, yes), family history of CRC (no, yes, possibly), history of hypertension (no, yes), history of diabetes (no, yes), history of colorectal diverticulitis/diverticulosis (no, yes), history of colorectal comorbidities (no, yes) and history of colorectal polyp (no, yes).

Subgroup analysis revealed no significant differences in the negative association between MQI and the mortality of CRC across various levels of independent variables within each subgroup. All interaction *p*-values were greater than 0.05, as detailed in Supplementary Table 3. Similar to the significant robust negative association observed between MQI value and CRC incidence rates, sensitivity analyses demonstrated a significant robust negative association between MQI value and CRC mortality rates (Table 5).

**Table 5:** The sensitivity analyses between MQI and CRC mortality.

Categories	HR^e^ (Quartile 4 vs Quartile 1, 95% CI)	p for trend
Exclude extreme energy intake^a^	0.55 (0.38,0.82)	0.001
Exclude extreme BMI^b^	0.60 (0.43,0.85)	0.001
Excluding patients diagnosed within 2 years	0.61 (0.44,0.86)	0.001
Replace the indicator of cigarettes smoked^c^	0.62 (0.45,0.87)	0.002
Excluding patients with diabetes, diverticulitis/diverticulosis or colorectal co-morbidity^d^	0.64 (0.45,0.91)	0.003

a Extreme energy intake was defined as energy intake >4,000 kcal/day or <500 kcal/day.

b BMI defined as body mass index at baseline (kg/m^2^).

c Adjusting for pack-years of smoking (continuous) instead of daily cigarette consumption (0, 1–20, or >20).

d Colorectal co-morbidity included Ulcerative colitis, Crohn’s disease, Gardner’s syndrome, or familial polyposis.

e HR was adjusted for age (years), sex (male, female), race (White and non-White), marital status (married, unmarried), smoking status (never, currently/ever), number of cigarettes smoked (0, 1–20, > 20 cigarettes/day), history of alcohol consumption (yes, no), history of colorectal diverticulitis/ diverticulosis (yes, no), history of colorectal comorbidities (yes, no), history of colorectal polyps (yes, no), body mass index (kg/m2), trail arm (intervention, control), aspirin use (yes, no), history of diabetes (yes, no), history of hypertension (yes, no) and family history of CRC (yes, no).

## Discussion

Based on data from the PLCO trial, this study examined the associations between MQI and CRC prognosis among 101,709 US adults. Higher MQI was associated with reduced CRC incidence and mortality overall. In subsite-specific analyses, significant inverse associations were observed between MQI and both incidence and mortality of distal colon cancer, as well as mortality of proximal colon cancer. These relationships remained statistically significant after adjusting for lifestyle, demographic, and clinical confounders. The RCS model further demonstrated linear relationships between MQI and CRC outcomes. Sensitivity analyses supported the robustness of these findings.

The relationship between dietary macronutrients and CRC risk has long been examined through a reductionist lens, focusing on isolated nutrients or single macronutrient classes while overlooking the interactive effects of carbohydrate, fat, and protein quality within whole dietary patterns (3–5). Emerging evidence underscores that the chemopreventive potential of diet hinges not on individual components, but on the synergistic interplay of macronutrient quality across food matrices (6, 7, 31). This paradigm shift is critical, as colorectal carcinogenesis is driven by multidimensional mechanisms including metabolic dysregulation, chronic inflammation, gut dysbiosis, and oxidative stress—all of which are modulated by the integrated quality of dietary exposures (32, 33). By prioritizing dietary patterns rich in low-glycemic carbohydrates, anti-inflammatory fats, and high-quality proteins, the MQI captures this complexity, offering a more holistic framework to evaluate dietary impacts on colorectal health (34, 35).

MQI’s protective effects against colorectal cancer likely stem from its ability to orchestrate metabolic, inflammatory, and microbial pathways (36). For instance, diets aligned with MQI principles—emphasizing whole grains, legumes, nuts, seafood, and plant-based fats—enhance insulin sensitivity and reduce postprandial glycemic variability, thereby mitigating hyperinsulinemia-driven cell proliferation (37, 38). Concurrently, these patterns attenuate chronic inflammation by suppressing pro-inflammatory cytokines and oxidative stress, which are implicated in DNA damage and tumor initiation (39, 40). The gut microbiota also plays a pivotal role, as MQI-optimized diets enrich butyrate-producing bacteria and reduce pro-carcinogenic bile acid metabolites, fortifying intestinal barrier integrity and inhibiting oncogenic signaling (41, 42). Furthermore, the abundance of antioxidants, polyphenols, and bioactive peptides in MQI-aligned foods synergistically combats free radical damage and promotes DNA repair (43, 44). Collectively, these mechanisms highlight MQI’s potential to reduce colorectal cancer incidence and mortality by rebalancing physiological homeostasis, underscoring the need for dietary guidelines that prioritize macronutrient quality in its entirety rather than isolated nutrient targets (45). While the current findings support the involvement of metabolic, inflammatory, and microbial pathways, future studies incorporating objective biomarkers—such as inflammatory markers (e.g., CRP, IL-6), insulin sensitivity indices (e.g., HOMA-IR), gut microbiome profiling (e.g., 16S rRNA sequencing), and metabolomic data—are needed to empirically validate and further elucidate the mechanistic role of MQI in colorectal carcinogenesis. Integrating multi-omics approaches in prospective cohorts would help clarify causal pathways and strengthen the biological plausibility of our observations.

The subgroup analysis uncovered a significant interaction (*p* < 0.05) between a history of colorectal polyps and the relationship between the MQI and the incidence of CRC. This finding is of great importance as it indicates that a history of colorectal polyps serves as an effect modifier for the protective effects of MQI. Specifically, our results highlight that a high MQI exhibits a more pronounced preventive efficacy against CRC in individuals without a history of polyps. This enhanced effect can be attributed to multiple biological mechanisms. Diets with high MQI scores are rich in dietary fiber and anti-inflammatory components, such as omega-3 polyunsaturated fatty acids (7). In populations without polyps, these dietary elements play a crucial role in maintaining intestinal barrier function and immune homeostasis more effectively (32). Moreover, individuals without a history of polyps generally have lower baseline inflammation levels. This lower baseline inflammation further amplifies the anti-inflammatory effects of a high-MQI diet, leading to a more significant suppression of NF-κB signaling and oxidative stress (31). Additionally, the greater metabolic plasticity observed in polyp-free individuals enables high-MQI diets to optimize insulin sensitivity and lipid metabolism more efficiently, thereby strengthening the cancer-preventive effects (15). These findings strongly emphasize the preventive potential of a high MQI prior to the development of precancerous lesions. They provide robust support for the development of precision prevention strategies that specifically target high-risk polyp-free populations, with the ultimate goal of maximizing the reduction in CRC incidence (33, 34).

Our findings reveal a distinct anatomical gradient in MQI’s protective effects against colorectal cancer, demonstrating significant inverse associations with mortality from both proximal and distal colon cancers, as well as incidence of distal colon cancer. This differential efficacy likely reflects heterogenous biological responses along the colorectal continuum. The distal colon’s unique physiological environment—characterized by higher transit time, greater fecal mutagen exposure, and distinct microbial metabolic activities—may amplify MQI’s chemopreventive mechanisms (45). Specifically, MQI’s emphasis on dietary fiber and anti-inflammatory nutrients could enhance detoxification processes in the distal segment by promoting fecal bulk and modulating pro-carcinogenic bacterial metabolites (32). Additionally, the proximal colon’s embryologic midgut origin and higher susceptibility to bile acid-induced DNA damage may create a distinct pathogenic landscape where MQI’s antioxidant and insulin-sensitizing properties play a more critical role in mortality reduction than in primary prevention (33, 34, 45). These observations underscore the need for site-specific prevention strategies that leverage MQI’s multi-targeted mechanisms across colorectal subsites (33).

This study presents several distinct strengths. Firstly, the data derive from a large-scale prospective cohort of over 100,000 participants with diverse occupational backgrounds across 10 screening centers in the United States, ensuring broad demographic and geographic representation. The extended follow-up period reinforces the reliability of the findings. Secondly, the prospective design of the PLCO study, coupled with sensitivity analyses, effectively mitigates reverse causation bias from subclinical conditions influencing dietary patterns, thereby enhancing the validity of observed associations. Rigorous adjustment for multiple potential confounders further strengthens the credibility of the results (15, 46). Most notably, this investigation represents the first systematic examination of the MQI in relation to CRC incidence, mortality, and anatomical subsite-specific outcomes, offering novel insights into dietary influences on CRC heterogeneity.

Several limitations require consideration. First, the dietary assessment was conducted only once, 3 years after baseline, which may introduce temporal bias. Given that dietary habits are subject to change over time, this single - time assessment could potentially underestimate the cumulative dietary effects on the development of colorectal cancer (47). Although the use of the abbreviated DHQ might somewhat limit the ability to capture the full variability of dietary intake, baseline assessments still offer a reasonable approximation of long - term dietary patterns, as supported by previous studies (22, 23, 26). Second, residual confounding from unmeasured variables remains an unavoidable issue, which is a common limitation in observational research. Despite our efforts to control for known confounders through statistical adjustments, there may still be unmeasured factors that could influence the relationship between macronutrient quality and colorectal cancer outcomes. Third, the reliance on self - reported dietary data via the DHQ is a significant concern. Recall bias is inevitable, as participants may not accurately remember their past dietary intake, leading to either under - or over - estimation. This inaccuracy in exposure measurement can affect the precision of our findings and may introduce bias into the results. Fourth, our findings are derived from a cohort predominantly composed of middle-aged and older White adults in the United States. Consequently, the generalizability of our results to younger populations or other ethnic and sociocultural groups may be limited. Future studies should validate these associations in more diverse populations, including different age, racial, and geographic cohorts. Finally, as with all observational studies, causal inferences regarding diet-CRC relationships must be interpreted cautiously, necessitating future randomized controlled trials to establish definitive causality.

## Conclusion

Considering the current evidence and the intricate interplay among dietary patterns, physical activity, and body composition, high-MQI dietary patterns should be viewed as an integral component of a healthy lifestyle rather than an isolated modifiable risk factor. In conclusion, this study establishes a significant association between elevated MQI and reduced incidence and mortality rates of colorectal cancer, particularly demonstrating heterogenous protective effects across anatomical subsites. These findings advance our understanding of precision nutrition strategies for CRC prevention, offering a robust evidence base for formulating tailored dietary recommendations and public health initiatives that synergistically integrate dietary quality improvement with other lifestyle modifications.

## Data Availability

The original contributions presented in the study are included in the article/Supplementary material, further inquiries can be directed to the corresponding authors.

## Footnotes

1
https://cdas.cancer.gov/learn/plco/early-qx/
