# Supplementary material for: Macronutrient quality and colorectal cancer outcomes: evidence from the PLCO screening trial
Source: Front Nutr. 2026 Jan 12;12:1656275. doi: 10.3389/fnut.2025.1656275 (PMC12832537; doi:10.3389/fnut.2025.1656275)
Supplement: Supplementary file 1 [file Table_1.docx]

**SUPPLEMENTARY MATERIAL**

**Macronutrient Quality and Colorectal Cancer Outcomes: Evidence from the PLCO Screening Trial.**

**Supplementary Table 1**. Distribution of variables with missing data before and after imputation.

| **Variable** | **Before imputation** | **After imputation** | **Number (%) with missing data** |
| --- | --- | --- | --- |
| **Race** |  |  | 37(0.04%) |
| white | 93986 (92.44%) | 94023 (92.44%) |  |
| non-white | 7686 (7.56%) | 7686 (7.56%) |  |
| **Marriage** |  |  | 193(0.19%) |
| married | 79595 (78.41%) | 79788 (78.45%) |  |
| unmarried | 21921 (21.59%) | 21921 (21.55%) |  |
| **Diabetes history** |  |  | 538(0.53%) |
| no | 94369 (93.28%) | 94907 (93.31%) |  |
| yes | 6802 (6.72%) | 6802 (6.69%) |  |
| **Aspirin use history** |  |  | 444(0.44%) |
| no | 53483 (52.81%) | 53927 (53.02%) |  |
| yes | 47782 (47.19%) | 47782 (46.98%) |  |
| **Family history of colorectal cancer** |  |  | 781(0.77%) |
| no | 88129 (87.32%) | 88910 (87.42%) |  |
| yes | 10306 (10.21%) | 10306 (10.13%) |  |
| possibly | 2493 (2.47%) | 2493 (2.45%) |  |
| **Diverticulitis/Diverticulosis history** |  |  | 643(0.64%) |
| no | 94243 (93.25%) | 94886 (93.29%) |  |
| yes | 6823 (6.75%) | 6823 (6.71%) |  |
| **Colorectal comorbidities history** |  |  | 897(0.89%) |
| no | 99456 (98.65%) | 100353 (98.67%) |  |
| yes | 1356 (1.35%) | 1356 (1.33%) |  |
| **Colorectal polyps history** |  |  | 626(0.62%) |
| no | 94318 (93.31%) | 94944 (93.35%) |  |
| yes | 6765 (6.69%) | 6765 (6.65%) |  |
| **Hypertension history** |  |  | 513(0.51%) |
| no | 68165 (67.36%) | 68678 (67.52%) |  |
| yes | 33031 (32.64%) | 33031 (32.48%) |  |
| **Family history of cancer** |  |  | 288(0.28%) |
| no | 44588 (43.96%) | 44876 (44.12%) |  |
| yes | 56833 (56.04%) | 56833 (55.88%) |  |
| **Smoking status** |  |  | 20(0.02%) |
| no | 48542 (47.74%) | 48562 (47.75%) |  |
| current/former | 53147 (52.26%) | 53147 (52.25%) |  |
| **Body mass index at baseline (kg/m2)** | 27.23±4.82 | 27.22±4.79 | 1348(1.34%) |
| **Weight fluctuation**^a^ | 2.88±0.76 | 2.88±0.76 | 1348(1.34%) |
| **Smoking pack-years** | 17.83±26.69 | 17.65±26.59 | 1163(1.16%) |
| **Daily cigarette consumption** |  |  | 124(0.12%) |
| 0 | 48542 (47.78%) | 48666 (47.85%) |  |
| 1-20 | 33203 (32.68%) | 33203 (32.65%) |  |
| >20 | 19840 (19.53%) | 19840 (19.51%) |  |

Note: Descriptive statistics are presented as (mean ± standard deviation) and number (percentage) for continuous and categorical.

^a^ Weight fluctuation defined as the participant's baseline weight minus weight at age 20.

**Supplementary Table 2**. Subgroup analyses between MQI and CRC incidence.

| **Variables** | **Number of participates** | | **Number of cases** | | **P_interaction_** |  | **HR ^b^ ( 95% confidence interval )** | | | **P_trend_ (Q4 vs Q1)** |
| --- | --- | --- | --- | --- | --- | --- | --- | --- | --- | --- |
|  |  |  |  |  |  | **Quartile 1** | **Quartile 2** | **Quartile 3** | **Quartile 4** |  |
| **Age(years)** |  |  | | 0.312 | |  |  |  |  |  |
| <=65 | 71,841 | 642 | |  | | 1.00 (reference) | 0.95 (0.78, 1.16) | 1.01 (0.82, 1.24) | 0.72 (0.57, 0.92) | 0.025 |
| >65 | 29,868 | 458 | |  | | 1.00 (reference) | 1.02 (0.80, 1.30) | 0.85 (0.66, 1.11) | 0.89 (0.69, 1.16) | 0.257 |
| **Sex** |  |  | | 0.136 | |  |  |  |  |  |
| male | 49,459 | 608 | |  | | 1.00 (reference) | 1.12 (0.92, 1.36) | 0.95 (0.76, 1.19) | 0.79 (0.62, 1.03) | 0.077 |
| female | 52,250 | 492 | |  | | 1.00 (reference) | 0.78 (0.61, 1.00) | 0.86 (0.68, 1.09) | 0.73 (0.57, 0.94) | 0.029 |
| **Race** |  |  | | 0.689 | |  |  |  |  |  |
| white | 94,023 | 1,003 | |  | | 1.00 (reference) | 0.98 (0.84, 1.15) | 0.94 (0.79, 1.12) | 0.79 (0.66, 0.95) | 0.016 |
| non-white | 7,686 | 97 | |  | | 1.00 (reference) | 0.77 (0.44, 1.34) | 0.71 (0.4, 1.24) | 0.66 (0.38, 1.16) | 0.161 |
| **Marriage** |  |  | | 0.920 | |  |  |  |  |  |
| married | 79,788 | 855 | |  | | 1.00 (reference) | 0.97 (0.82, 1.16) | 0.94 (0.78, 1.14) | 0.79 (0.65, 0.97) | 0.030 |
| non-married | 21,921 | 245 | |  | | 1.00 (reference) | 0.95 (0.68, 1.33) | 0.85 (0.60, 1.20) | 0.75 (0.52, 1.08) | 0.097 |
| **Hypertension history** |  |  | | 0.665 | |  |  |  |  |  |
| no | 68,678 | 749 | |  | | 1.00 (reference) | 0.91 (0.75, 1.10) | 0.87 (0.71, 1.06) | 0.77 (0.62, 0.95) | 0.014 |
| yes | 33,031 | 351 | |  | | 1.00 (reference) | 1.09 (0.84, 1.43) | 1.04 (0.78, 1.39) | 0.79 (0.57, 1.10) | 0.218 |
| **Diabetes history** |  |  | | 0.208 | |  |  |  |  |  |
| no | 94,907 | 994 | |  | | 1.00 (reference) | 0.96 (0.82, 1.14) | 0.90 (0.75, 1.07) | 0.81 (0.67, 0.97) | 0.018 |
| yes | 6,802 | 106 | |  | | 1.00 (reference) | 0.97 (0.60, 1.58) | 1.12 (0.69, 1.82) | 0.47 (0.23, 0.94) | 0.097 |
| **Smoking status** |  |  | | 0.622 | |  |  |  |  |  |
| current/former | 53,147 | 615 | |  | | 1.00 (reference) | 1.03 (0.84, 1.26) | 0.92 (0.74, 1.15) | 0.73 (0.57, 0.94) | 0.014 |
| no | 48,562 | 485 | |  | | 1.00 (reference) | 0.90 (0.71, 1.14) | 0.93 (0.73, 1.19) | 0.83 (0.64, 1.07) | 0.190 |
| **Colorectal polyps history** |  |  | | 0.035 | |  |  |  |  |  |
| no | 94,944 | 1,006 | |  | | 1.00 (reference) | 0.93 (0.79, 1.10) | 0.96 (0.81, 1.14) | 0.78 (0.65, 0.94) | 0.017 |
| yes | 6,765 | 94 | |  | | 1.00 (reference) | 1.39 (0.85, 2.28) | 0.50 (0.26, 0.99) | 0.80 (0.44, 1.45) | 0.138 |
| **Colorectal comorbidities history** |  |  | | 0.173 | |  |  |  |  |  |
| no | 100,353 | 1,084 | |  | | 1.00 (reference) | 0.95 (0.81, 1.11) | 0.93 (0.79, 1.09) | 0.79 (0.66, 0.94) | 0.009 |
| yes | 1,356 | 16 | |  | | 1.00 (reference) | 3.23(1.00,10.48) | 0.60 (0.11, 3.22) | 0.38 (0.04, 3.38) | 0.241 |
| **Diverticulitis/Diverticulosis history** |  |  | | 0.119 | |  |  |  |  |  |
| no | 94,886 | 1,022 | |  | | 1.00 (reference) | 0.92 (0.78, 1.08) | 0.90 (0.76, 1.06) | 0.76 (0.63, 0.92) | 0.004 |
| yes | 6,823 | 78 | |  | | 1.00 (reference) | 2.05 (1.13, 3.71) | 1.46 (0.77, 2.78) | 1.19 (0.59, 2.41) | 0.827 |
| **BMI**^a^ |  |  | | 0.326 | |  |  |  |  |  |
| <=30 | 78,562 | 833 | |  | | 1.00 (reference) | 0.93 (0.77, 1.11) | 0.88 (0.73, 1.06) | 0.79 (0.65, 0.97) | 0.034 |
| >30 | 23,147 | 267 | |  | | 1.00 (reference) | 1.07 (0.80, 1.44) | 1.02 (0.73, 1.41) | 0.59 (0.38, 0.93) | 0.065 |
| **Aspirin use history** |  |  | | 0.382 | |  |  |  |  |  |
| no | 53,927 | 605 | |  | | 1.00 (reference) | 1.02 (0.83, 1.25) | 0.94 (0.75, 1.17) | 0.71 (0.55, 0.90) | 0.006 |
| yes | 47,782 | 495 | |  | | 1.00 (reference) | 0.89 (0.71, 1.13) | 0.90 (0.70, 1.15) | 0.87 (0.67, 1.12) | 0.269 |
| **Family history of colorectal cancer** |  |  | | 0.200 | |  |  |  |  |  |
| no | 88,910 | 937 | |  | | 1.00 (reference) | 0.97 (0.82, 1.14) | 0.85 (0.71, 1.02) | 0.77 (0.63, 0.93) | 0.003 |
| yes/possibly | 12,799 | 163 | |  | | 1.00 (reference) | 0.96 (0.63, 1.46) | 1.35 (0.91, 2.01) | 0.83 (0.52, 1.34) | 0.857 |
| **Daily cigarette consumption** |  |  | | 0.809 | |  |  |  |  |  |
| 0 | 48,666 | 491 | |  | | 1.00 (reference) | 0.91 (0.72, 1.16) | 0.93 (0.73, 1.19) | 0.84 (0.65, 1.09) | 0.213 |
| 1-20 | 33,203 | 377 | |  | | 1.00 (reference) | 1.02 (0.79, 1.33) | 1.01 (0.77, 1.33) | 0.73 (0.54, 1.00) | 0.080 |
| >20 | 19,840 | 232 | |  | | 1.00 (reference) | 1.02 (0.74, 1.39) | 0.76 (0.52, 1.12) | 0.72 (0.47, 1.09) | 0.063 |
| **History of alcohol consumption** |  |  | | 0.899 | |  |  |  |  |  |
| no | 27,741 | 291 | |  | | 1.00 (reference) | 1.02 (0.75, 1.37) | 0.94 (0.68, 1.30) | 0.79 (0.56, 1.11) | 0.163 |
| yes | 73,968 | 809 | |  | | 1.00 (reference) | 0.95 (0.79, 1.14) | 0.92 (0.76, 1.11) | 0.78 (0.63, 0.96) | 0.019 |

^a^ BMI was defined as body mass index at baseline (kg/m2).

^b^ Hazard ratio was adjusted for age (years), sex (male, female), race (white and non-white), marital status (married, unmarried), smoking status (never, currently/ever), number of cigarettes smoked (0, 1-20, > 20 cigarettes/day), history of alcohol consumption (yes, no), history of colorectal diverticulitis/ diverticulosis (yes, no), history of colorectal comorbidities (yes, no), history of colorectal polyps (yes, no), body mass index (kg/m2), trail arm (intervention, control), aspirin use (yes, no), history of diabetes (yes, no), history of hypertension (yes, no) and family history of CRC (yes, no).

**Supplementary Table 3**. Subgroup analyses between MQI and CRC mortality.

| **Variables** | **Number of participates** | | **Number of cases** | | **P_interaction_** |  | **HR ^b^ ( 95% confidence interval )** | | | **P_trend_ (Q4 vs Q1)** |
| --- | --- | --- | --- | --- | --- | --- | --- | --- | --- | --- |
|  |  |  |  |  |  | **Quartile 1** | **Quartile 2** | **Quartile 3** | **Quartile 4** |  |
| **Age(years)** |  |  | | 0.318 | |  |  |  |  |  |
| <=65 | 71,841 | 168 | |  | | 1.00 (reference) | 0.75 (0.50, 1.12) | 0.82 (0.54, 1.24) | 0.64 (0.40, 1.02) | 0.066 |
| >65 | 29,868 | 146 | |  | | 1.00 (reference) | 0.88 (0.59, 1.32) | 0.53 (0.32, 0.87) | 0.63 (0.39, 1.02) | 0.017 |
| **Sex** |  |  | | 0.721 | |  |  |  |  |  |
| male | 49,459 | 182 | |  | | 1.00 (reference) | 0.89 (0.62, 1.27) | 0.61 (0.39, 0.95) | 0.62 (0.38, 0.99) | 0.012 |
| female | 52,250 | 132 | |  | | 1.00 (reference) | 0.69 (0.43, 1.10) | 0.70 (0.44, 1.12) | 0.61 (0.38, 0.98) | 0.049 |
| **Race** |  |  | | 0.997 | |  |  |  |  |  |
| white | 94,023 | 281 | |  | | 1.00 (reference) | 0.80 (0.59, 1.08) | 0.66 (0.47, 0.93) | 0.61 (0.42, 0.87) | 0.002 |
| non-white | 7,686 | 33 | |  | | 1.00 (reference) | 0.81 (0.32, 2.07) | 0.62 (0.23, 1.67) | 0.71 (0.27, 1.84) | 0.441 |
| **Marriage** |  |  | | 0.691 | |  |  |  |  |  |
| married | 79,788 | 230 | |  | | 1.00 (reference) | 0.86 (0.62, 1.19) | 0.64 (0.43, 0.93) | 0.66 (0.45, 0.98) | 0.014 |
| non-married | 21,921 | 84 | |  | | 1.00 (reference) | 0.65 (0.36, 1.16) | 0.70 (0.39, 1.25) | 0.50 (0.26, 0.95) | 0.037 |
| **Hypertension history** |  |  | | 0.737 | |  |  |  |  |  |
| no | 68,678 | 210 | |  | | 1.00 (reference) | 0.75 (0.53, 1.06) | 0.59 (0.40, 0.88) | 0.52 (0.34, 0.79) | 0.001 |
| yes | 33,031 | 104 | |  | | 1.00 (reference) | 0.92 (0.56, 1.51) | 0.82 (0.47, 1.41) | 0.85 (0.48, 1.50) | 0.490 |
| **Diabetes history** |  |  | | 0.247 | |  |  |  |  |  |
| no | 94,907 | 285 | |  | | 1.00 (reference) | 0.78 (0.58, 1.06) | 0.60 (0.42, 0.85) | 0.63 (0.44, 0.89) | 0.002 |
| yes | 6,802 | 29 | |  | | 1.00 (reference) | 0.98 (0.37, 2.59) | 1.33 (0.53, 3.30) | 0.42 (0.11, 1.59) | 0.356 |
| **Smoking status** |  |  | | 0.902 | |  |  |  |  |  |
| current/former | 53,147 | 180 | |  | | 1.00 (reference) | 0.81 (0.56, 1.17) | 0.61 (0.40, 0.94) | 0.54 (0.34, 0.87) | 0.003 |
| no | 48,562 | 134 | |  | | 1.00 (reference) | 0.79 (0.50, 1.24) | 0.73 (0.45, 1.18) | 0.72 (0.44, 1.16) | 0.154 |
| **Colorectal polyps history** |  |  | | 0.273 | |  |  |  |  |  |
| no | 94,944 | 284 | |  | | 1.00 (reference) | 0.75 (0.55, 1.01) | 0.67 (0.48, 0.93) | 0.58 (0.41, 0.83) | 0.001 |
| yes | 6,765 | 30 | |  | | 1.00 (reference) | 1.45 (0.59, 3.61) | 0.59 (0.18, 1.95) | 1.09 (0.39, 3.03) | 0.813 |
| **Colorectal comorbidities history** |  |  | | 0.518 | |  |  |  |  |  |
| no | 100,353 | 310 | |  | | 1.00 (reference) | 0.79 (0.59, 1.05) | 0.66 (0.48, 0.91) | 0.62 (0.45, 0.87) | 0.002 |
| yes | 1,356 | 4 | |  | | 1.00 (reference) | 1.51(0.08,27.72) | 0.51(0.02,13.35) | / | 0.287 |
| **Diverticulitis/Diverticulosis history** |  |  | | 0.186 | |  |  |  |  |  |
| no | 94,886 | 296 | |  | | 1.00 (reference) | 0.75 (0.55, 1.00) | 0.62 (0.45, 0.87) | 0.61 (0.43, 0.86) | 0.001 |
| yes | 6,823 | 18 | |  | | 1.00 (reference) | 2.88(0.83,10.01) | 1.96 (0.52, 7.46) | 0.89 (0.16, 5.05) | 0.889 |
| **BMI**^a^ |  |  | | 0.598 | |  |  |  |  |  |
| <=30 | 78,562 | 232 | |  | | 1.00 (reference) | 0.74 (0.53, 1.04) | 0.60 (0.41, 0.87) | 0.55 (0.37, 0.80) | 0.001 |
| >30 | 23,147 | 82 | |  | | 1.00 (reference) | 0.95 (0.55, 1.63) | 0.85 (0.45, 1.58) | 0.82 (0.41, 1.63) | 0.501 |
| **Aspirin use history** |  |  | | 0.207 | |  |  |  |  |  |
| no | 53,927 | 171 | |  | | 1.00 (reference) | 0.95 (0.66, 1.38) | 0.73 (0.47, 1.12) | 0.52 (0.32, 0.86) | 0.006 |
| yes | 47,782 | 143 | |  | | 1.00 (reference) | 0.63 (0.40, 0.98) | 0.59 (0.36, 0.95) | 0.71 (0.45, 1.13) | 0.091 |
| **Family history of colorectal cancer** |  |  | | 0.148 | |  |  |  |  |  |
| no | 88,910 | 265 | |  | | 1.00 (reference) | 0.78 (0.57, 1.06) | 0.61 (0.43, 0.87) | 0.67 (0.47, 0.95) | 0.008 |
| yes/possibly | 12,799 | 49 | |  | | 1.00 (reference) | 0.91 (0.45, 1.81) | 0.91 (0.44, 1.89) | 0.26 (0.08, 0.90) | 0.046 |
| **Daily cigarette consumption** |  |  | | 0.961 | |  |  |  |  |  |
| 0 | 48,666 | 136 | |  | | 1.00 (reference) | 0.78 (0.50, 1.22) | 0.75 (0.47, 1.20) | 0.71 (0.44, 1.14) | 0.147 |
| 1-20 | 33,203 | 98 | |  | | 1.00 (reference) | 0.94 (0.57, 1.54) | 0.67 (0.38, 1.18) | 0.59 (0.32, 1.09) | 0.055 |
| >20 | 19,840 | 80 | |  | | 1.00 (reference) | 0.69 (0.39, 1.21) | 0.51 (0.25, 1.01) | 0.51 (0.25, 1.06) | 0.024 |
| **History of alcohol consumption** |  |  | | 0.877 | |  |  |  |  |  |
| no | 27,741 | 87 | |  | | 1.00 (reference) | 0.88 (0.52, 1.50) | 0.63 (0.34, 1.17) | 0.59 (0.31, 1.11) | 0.061 |
| yes | 73,968 | 227 | |  | | 1.00 (reference) | 0.77 (0.55, 1.08) | 0.68 (0.47, 0.98) | 0.62 (0.42, 0.93) | 0.010 |

^a^ BMI was defined as body mass index at baseline (kg/m2).

^b^ Hazard ratio was adjusted for age (years), sex (male, female), race (white and non-white), marital status (married, unmarried), smoking status (never, currently/ever), number of cigarettes smoked (0, 1-20, > 20 cigarettes/day), history of alcohol consumption (yes, no), history of colorectal diverticulitis/ diverticulosis (yes, no), history of colorectal comorbidities (yes, no), history of colorectal polyps (yes, no), body mass index (kg/m2), trail arm (intervention, control), aspirin use (yes, no), history of diabetes (yes, no), history of hypertension (yes, no) and family history of CRC (yes, no).
